# Influence of Chitosan and Grape Seed Extract on Thermal and Mechanical Properties of PLA Blends

**DOI:** 10.3390/polym15061570

**Published:** 2023-03-22

**Authors:** Victoria Goetjes, Claudia L. von Boyneburgk, Hans-Peter Heim, Marilia M. Horn

**Affiliations:** 1 Institute of Material Engineering, Polymer Engineering, University of Kassel, Mönchebergstr. 3, 34125 Kassel, Germany; 2 Physical Chemistry of Nanomaterials, Institute of Chemistry and Center for Interdisciplinary Nanostructure Science and Technology (CINSaT), University of Kassel, Heinich-Plett Straße 40, 34109 Kassel, Germany

**Keywords:** chitosan, PLA, grape seed extract, DPPH assay

## Abstract

Blends based on polylactic acid (PLA), chitosan, and grape seed extract (GE) were prepared by extrusion and injection molding. The effect of chitosan (5% and 15% on PLA basis) and natural extract (1% on PLA basis) incorporated into the PLA host matrix was explored regarding the thermal and mechanical properties. GE showed antioxidant activity, as determined by the DPPH assay method. Chitosan and GE affect the degree of crystallinity up to 30% as the polysaccharide acts as a nucleating agent, while the extract reduces the mobility of PLA chains. The decomposition temperature was mainly affected by adding chitosan, with a reduction of up to 25 °C. The color of the blends was specially modified after the incorporation of both components, obtaining high values of b* and L* after the addition of chitosan, while GE switched to high values of a*. The elongation at break (EB) exhibited that the polysaccharide is mainly responsible for its reduction of around 50%. Slight differences were accessed in tensile strength and Young’s modulus, which were not statistically significant. Blends showed increased irregularities in their surface appearance, as observed by SEM analysis, corresponding to the partial miscibility of both polymers.

## 1. Introduction

Sustainable development has become a demand in modern society due to environmental concerns and the concomitant decrease in fossil resources. Polymers are an inherent part of our daily life. Renewable sources provide polymers for the replacement of conventional fossil-based raw materials aiming at sustainable development [1]. Research shows that biopolymers can be manufactured by blending two polymers to develop new materials that exhibit properties that could not be achieved by using each individual polymer. In addition, the nanostructure of that blend can be modified by adding additive material, which will enhance desirable properties.

Thermoplastic polymers exhibit low process temperatures compared to thermosets, which is advantageous for the development of packaging materials. Polylactic acid (PLA) is one of the most promising thermoplastics, as it is considered a fully sustainable and biodegradable polymer [2]. Its production is based on the fermentation of dextrose, which is converted into lactic acid, followed through polymerization. After disposal, the PLA-based materials are degraded into the water, and carbon dioxide is consumed to grow more agricultural products [3]. Nevertheless, toughness and strong hydrophobic properties limit PLA for packaging and biomedical applications. Strategies to overcome this limitation are usually related to employing methods such as copolymerization [4] or the addition of fillers [5]. Nevertheless, the copolymerization process involves the use of chemicals or generates unsafe subproducts. Then, a sustainable approach in the development of ecofriendly PLA materials includes the use of reinforcement materials, such as flax fibers [6], cellulose nanofiber [7], silk fibroin [8], or montmorillonite clay [9]. Alternatively, simply blending with another polymer is one of the most effective solutions. Pradeep et al. [10] investigated the use of PLA/poly (butylene succinate-co-adipate) (PBSA) and observed an increment in the crystallinity of the composites. In a study of blend filaments of PCL/PLA, the authors showed the effect on tensile strength regarding the concentration of PCL [11]. In our study, the aim was to combine PLA with a biopolymer that should provide appropriate mechanical and morphological properties without losing its biodegradable behavior. In that concern, chitosan is a polymer that fits those conditions.

Chitosan is obtained from the chitin deacetylation process in the presence of a strong alkali [12]. Besides its biodegradability properties, chitosan is considered a renewable and green polymer, as it is derived from waste sources in the fish industry [13]. In this regard, this polysaccharide also has promising potential for the packaging industry, improving hydrophilic capacity and adding ductility to the blends.

The association of both renewable polymers has already been described in the literature. Suyatma et al. [14] described the preparation of PLA/chitosan films by the casting process and observed an increase in both tensile strength and elongation at break associated with the rise of chitosan concentration. Bonilla et al. [15] studied the PLA/chitosan mixture by extrusion, but only a low amount of the polysaccharide was considered in the research. Claro et al. [16] compared PLA/chitosan and PLA/cellulose acetate films and found that plasticizers are not necessary to produce blends with potential use applications.

Our study addresses the development of PLA and chitosan blends using the extrusion process and injection molding process, which is known as an efficient technique for incorporating chitosan into PLA host matrixes [15]. Additionally, the traditional casting method for film preparation could not be advantageous, as solvent residues can be left in the final product due to the difficulty of controlling the solvent evaporation process [17].

Grape seed extract mainly consists of flavonoids, tocopherol, and other phenolic compounds responsible for its antioxidant activity [18]. Then, the grape seed extract was chosen as the additive active material of the blends. Recently, Wang et al. [17] described the preparation of films of poly(ε-caprolactone)/chitosan loaded with grapefruit seed extract and found an application as a packaging material. Nevertheless, poly(ε-caprolactone) is not considered a renewable source polymer. At this point, our contribution is to describe a blend composed of two renewable and biodegradable polymers (PLA and chitosan) associated with an additive (grape seed extract) to improve the antioxidant features without compromising the mechanical and thermal characteristics of these blends.

## 2. Materials and Methods

### 2.1. Materials

Polylactic acid (PLA) (grade: 3052D Ingeo, L-lactic acid/D-lactic acid: 96/4 and MM = 116,000 g mol^−1^) used in this study was supplied by NatureWorks LLC, Plymouth, MA, USA. Low-molecular-weight chitosan was purchased from Sigma Aldrich (DA 15% and MM 120,000 g mol^−1^). The grape seed extract was obtained from Buxtrade GmbH (Germany), and it mainly contained oligomeric proanthocyanidins, as specified by the supplier.

### 2.2. Antioxidant Properties of Grape Seed Extract

The free radical scavenging activity of the grape seed extract was evaluated by 1,1-diphenyl-2-picryl-hydrazil (DPPH) following the method adapted from [18]. The DPPH solution was prepared in ethanol and mixed with a diluted extract solution. The volume was adjusted with ethanol to a final volume of 5 mL. After incubation in the dark for 30 min at room temperature, the absorbance was measured at 517 nm against ethanol as blank using a UV Perkin Elmer Lambda 900. The scavenging activity of the sample was compared with a control (DPPH solution and ethanol). The absorbance of the control and the extract sample was measured in triplicate, and the activity was calculated according to Equation (1).

(1) $$DPPH\;scavening\;activity\;\left(\%\right)=\left[\frac{\left(A_{c}-A_{s}\right)}{A_{c}}\right]\times 100$$

where *A_c_* is the absorbance of the control solution and *A_s_* is the absorbance of the extract sample solution.

### 2.3. Extrusion

PLA granules and chitosan powder were dried at 40 °C overnight to remove the absorbed moisture. Both biopolymers were mixed in a beaker in different ratios of PLA/CHI (95/05 and 85/15), namely P95C5 and P85C15, respectively. A control sample of PLA was prepared to allow for a comparison effect of chitosan addition in the binary blends. Blends containing the dye molecule, grape seed extract, were prepared following the same ratio of PLA and chitosan. A fixed amount of 10 mg was used in our study. Then, samples were labeled as PLAGE, P95C5GE, and P85C15GE, where the suffix “GE” means the addition of the grape seed extract.

Each mixture was extruded in a twin-screw extruder (Haake Rheomix, ThermoFischer, Dreieich, Germany), and the screw speed and processing temperature were maintained at 50 rpm and 160 °C, respectively. The recycle time was set up in 5 min. The extruded strips were collected and stored in a humidity control chamber (50% and 23 °C).

### 2.4. Differential Scanning Calorimetry (DSC)

The thermal properties of PLA and the extruded samples with or without grape seed extract were determined by differential scanning calorimetry (Perkin-Elmer DSC 7). The glass transition temperature (T_g_) and the intrinsic degree of crystallinity (*X_c_*) as a function of the composition changes (chitosan and grape seed extract addition). The samples were heated from 20 to 200 °C at a rate of 5 °C min^−1^. All sample weights were between 6 and 8 mg.

The onset temperature of the glass transition was determined as T_g_ in the software of the equipment. The *X_c_* (%) was calculated from Equation (2):

(2) $$Xc\left(\%\right)=\frac{∆Hm}{w\times ∆H{}^{\circ}m}\times 100$$

where Δ*H_m_* is the measured melting enthalpy, w is the mass fraction of PLA in the composite, and Δ*H°_m_* is the corresponding enthalpy of 100% PLA crystalline polymer (93 J g^−1^).

### 2.5. Thermogravimetry (TGA)

TGA analyses were performed on a Perkin-Elmer, Pyris Diamond TG/DTA. Thermogravimetric curves were performed under a synthetic air atmosphere. Samples of approximately 8 mg were loaded into a platinum crucible and heated from 25 to 800 °C at a heating rate of 10 °C min^−1^.

### 2.6. Tensile Tests

Using the extrudate produced as previously described, shoulder bars of type 5A were manufactured with the aid of a miniature injection molding machine (Haake MiniJet, ThermoFischer, Dreieich, Germany). Tensile tests were carried out using a Zwick Z010 from Zwick & Roell according to the standard DIN EN ISO 527 at a test speed of 2 mm/min. Tensile strength, maximum elongation at break, and Young’s modulus were evaluated. At least three measurements with each blend (17.5 × 4 mm) were performed. Tensile strength (TS) was expressed in megapascals (MPa) and calculated by dividing the maximum load (N) by the specimen’s initial cross-sectional area (m^2^). EB was determined by the ratio of the final length (sample rupture) to the initial length of the specimen and expressed as a percentage. Young’s modulus was evaluated by the slope of the linear portion of the stress–strain curve.

Tensile test results were statistically treated using analysis of variance (ANOVA) and Tukey’s test with a significance level set at 5%. The test was performed with the Origin software and results were presented as the mean value and the standard deviation.

### 2.7. Color Measurements

The injection-molded test specimens were subjected to color spectroscopy. Color changes were examined by spectroscopy, based on the CIE L*, a*, and b* color space, in which L* represents the lightness from 0 (black) to 100 (white), a* express green/red, and b* depict the yellow–blue dimension. The measurement was performed on an Ultra Scan Pro (Hunterlab, Reston, VA, USA) in a replicate assessment. The results were plotted as a function of the color space diagram.

### 2.8. Scanning Electron Microscopy (SEM)

The morphology of the blends was examined using scanning electron microscopy (SEM). Approximately 0.5 cm^2^ sample size was placed in the stubs and covered using a thin layer of palladium-gold to improve their conductivity properties. The coating was performed in a Sputter coater from Polaron, and a thickness of 10 nm was deposited. The SEM images were acquired in an S-4000 equipment from Hitachi.

## 3. Results and Discussion

### 3.1. DHPP Antioxidant Assay

Grape seeds are the main by-products of juice and wine production, considered a waste material. Nevertheless, they consist of approximately 60–70% of polyphenols in their composition, a valuable compound with potent antioxidant activity by effectively scavenging free radicals. The free radical scavenging activity of the grape seed extract was tested using the DPPH method through the change of absorbance due to the reduction in the DPPH radical.

The inhibition percentage of the DPPH radical, based on the concentration used in the blends, was 78.12 ± 5.03%. A previous study described values ranging from 83% and 99% [10], in which the values differ due to the method of extraction, the species of grape, and even the season of cultivation [19]. The antioxidant activity of the grape seed extract was shown to be effective, and its addition to the blends improves their functionality against free radicals.

### 3.2. Differential Scanning Calorimetry (DSC)

Extrusion is a convenient method to prepare environmentally friendly polymer blends without using an organic solvent. Additionally, it is an efficient way to improve the blending of the components and prevent the formation of phase separation.

**Figure 1:**
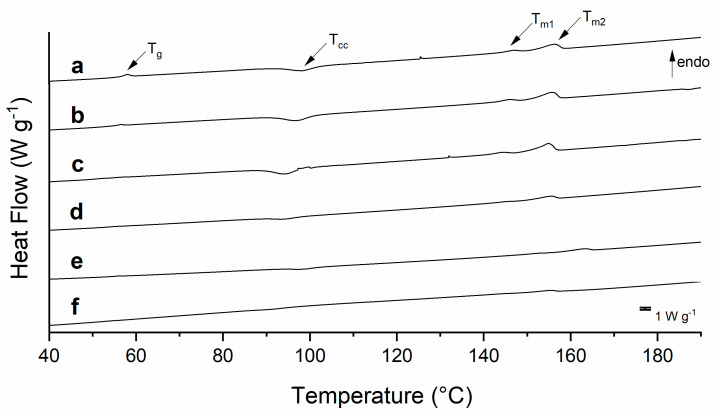
DSC curves for (a) PLA; (b) P95c5; (c) P85C15; (d) PLAGE; (e) P95C5GE; and (f) P85C15GE.

As the PLA is the main compound of the blends, we want to analyze the effect of chitosan and grape seed extract addition on its crystallinity degree (Figure 1). Moreover, the glass transition temperature (T_g_) is a practical criterion for analyzing the miscibility of the components. Indeed, the T_g_ value should be affected by the partial miscibility of the components, and it is usually composition-dependent.

Semicrystalline polymers, such as PLA, under measurement conditions followed in this study, show endothermic peaks related to the glass transition temperature (T_g_) and melting (T_m_). The glass transition (PLA, T_g_) is found to be 58.6 °C, which is a comparable value reported by [20]. The exothermic peak at 98.2 °C refers to the crystallization temperature (T_cc_), a thermal event observed after the glass transition in semicrystalline polymers. In fact, after the Tg, there is an orientation and order in the PLA chains due to the increase in mobility during the heating process, which is reflected in the crystallization process. A flat heat flow in the cooling step of the curve is observed (not shown), which means the PLA does not crystallize during the cooling ramp at 10 °C min^−1^. There are two main reasons to explain this behavior. First, a portion of the D isomer inhibits the organization and crystallization of PLA chains. Second, the high cooling rate does not give enough time for the PLA chains to rearrange and organize in a crystalline structure.

Additionally, two peaks observed at 147.3 °C and 156.3 °C are associated with double melting peaks for PLA [21], which is attributed to the melting of two kinds of PLA crystals with different lamellae thicknesses [22].

No significant modification of the glass transition is observed for the low-weight fraction of chitosan (Figure 1b), as a slight reduction of around 2 °C is noticed. However, sample P85C15 (Figure 1c) exhibits two peaks in the glass transition region (highlighted with * in Table 1), which confirms the phase separation of both components. Bonilla et al. [15] studied PLA/CHI ratios of 95:5 and 90:10 and did not observe any lack of miscibility of both polymers. Our study shows that increasing the chitosan ratio (P85C15) leads to phase separation. The cold crystallization temperature (Tc) decreases with the addition of chitosan, which means that chitosan acts as a nucleation agent that favors PLA crystallization. This observation agrees with the study by [23] on the thermal behavior of PLA and PLA/natural rubber. Similarly, the polymer at a low weight fraction allows a heterogeneous nucleation mechanism of the PLA, reducing the free energy barrier and fastening the crystallization process.

Comparably to the neat PLA sample, the blends show the two melting peaks (T_m1_ and T_m2_) due to the recrystallization process observed during heating. Then, the peak at a lower temperature is attributed to the reorganization of the less crystalline structure, while the Tm_2_ refers to the more perfect crystalline structure of PLA [22]. The total melting enthalpy (Δ*H_m_*) of this thermal event was applied in Equation (1) to calculate the degree of crystallinity (*X_c_*) of the samples (Table 1).

With the addition of the grape seed extract, the glass transition peak was shifted to lower temperatures compared to the transition temperature observed for neat PLA (Figure 1d) and blends without the extract (Figure 1e,f). Additionally, it affects the cold crystallization temperature and reduces the enthalpy involved in the thermal event. A reduction in the Δ*H_cc_* might be related to a decrease in the recrystallization process after the glass transition event. Thus, chitosan and grape seed extract affect the degree of crystallization of the blends, as they act as a hindrance to disrupting the PLA crystalline sites.

A degree of crystallinity (*X_c_*) of 25.8% was found for the pure PLA (Table 1), a comparable value found in the literature [20]. Interestingly, chitosan increases the *X_c_* mainly due to the phase separation observed in the sample P85C15, allowing the PLA to dense pack in a crystalline form, as the polysaccharide acts as a nucleating agent. The grape extract addition reduces the crystallinity of PLAGE compared to the control sample, which is related to the reduced mobility of PLA chains. In fact, even low-weight contents of additives change the crystallization, and consequently the heat involved in the process, as observed by Yu et al., with the addition of Talc in PLA filaments [24].

### 3.3. Thermogravimetry (TGA)

**Figure 2:**
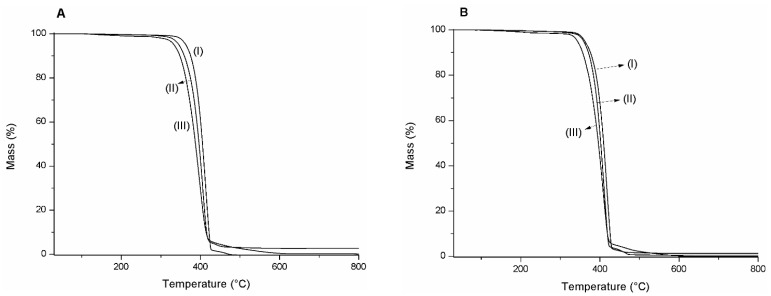
TGA curves. In (**A**): (I) PLA, (II) P95C5, and (III) P85C15. In (**B**), (I) PLAGE, (II) P95C5GE, and (III) P85C15GE.

The weight loss curves of pure PLA and blends are reported in Figure 2. A complete weight loss in a single step was observed for pure PLA, with an onset temperature of thermal decomposition at 318.7 °C (Table 2).

The onset temperature is the beginning of weight loss and reflects the thermal stability of the material. As PLA is the main compound in the polymeric blends, the inclusion of chitosan did not change the single-step profile; even then, chitosan is characterized by two significant degradation steps [25]. The single weight loss is associated with the relatively low chitosan ratio in the blends. Nevertheless, chitosan addition reduces the decomposition temperature up to 10 °C for the blend with a low amount of polysaccharide (5%) to around 25 °C for the blend with a high content (15%).

In general, the presence of grape seed extract does not change the thermal properties observed for PLA/chitosan ones, as comparable degradation temperatures were obtained. Lately, the low ratio of the extract could explain the behavior.

### 3.4. Tensile Tests

The average values and standard deviation of the mechanical properties of the specimens prepared in this study are reported in Table 3.

**Figure 3:**
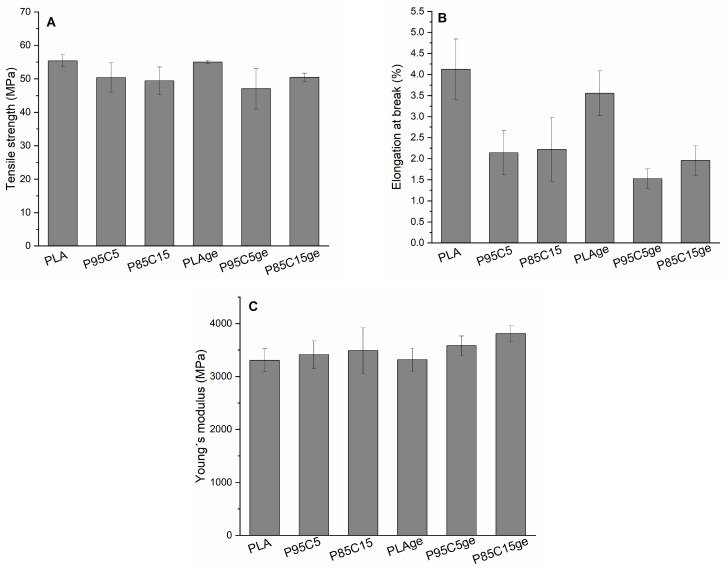
Mechanical properties of PLA and blends measured by tensile tests. In (**A**), tensile strength; in (**B**), elongation at break; in (**C**), Young´s modulus results.

The effect on tensile strength (TS) of chitosan and grape seed extract incorporated in PLA-based injection-molded shoulder bars was observed as a function of the chitosan loading of 5% and 15% (*w*/*w*) and the addition of 1% of the GE regarding the dry weight of biopolymers (Figure 3A). The TS of the pure PLA specimen was found to be 55.4 ± 1.8 MPa, which means a very brittle behavior, a value in agreement with the literature [26]. Adding chitosan decreases the TS in the binary blends, displaying values up to 50.4 ± 4.4 MPa (P95C5) and 49.4 ± 4.1 MPa (P85C15). Even though a slight decrease in TS was noticed, the change is not statistically significant. In the same way, no change in tensile strength was observed after incorporating the grape seed extract into the neat PLA sample. Nevertheless, comparing this set of samples, the introduction of the extract reduces the TS values in P85C15GE (50.5 ± 1.2 MPa) and P95C5GE (47.0 ± 6.0 MPa).

The elongation at break (EB) exhibited that the inclusion of chitosan is the main responsible for its reduction (Figure 3B). Indeed, all the samples containing the polysaccharide showed a reduction of around 50% in the EB values. In fact, the simple fact of adding the grape seed extract does not change the EB by comparing PLA and PLAGE values. However, the inclusion of chitosan showed that the reduction was significant, as confirmed by the statistical analysis.

The behavior of tensile properties in composites is very sensitive due to interfacial adhesion in composite mixtures. In fact, strength and elongation at break decrease when interfacial affinity is not ensured [27]. Our results established that adding chitosan imparted mechanical properties due to the decrease in both analyzed features. Other authors observed the same characteristic when cellulose in different forms was combined with PLA. Sanchez-Garcia and Lagaron described the same reduction in mechanical properties by the mixing of PLA and cellulose nanowhiskers, while Petersson and Oksman showed a reduction in elongation at a break of about 16% when microcrystalline cellulose was used as reinforcement of PLA. Even though Zakaria et al. reported an increase in tensile strength when 5% chitosan was used in PLA–chitosan blends, a further increase in the polysaccharide concentration implied a reduction in the tensile strength [28]. Nevertheless, the reinforcement of PLA was intrinsically related to a better dispersion of the guest material and also the compatibility between the components, mainly defined by the interfacial affinity.

Young’s modulus is one of the most important structural parameters of materials, determined by the coefficient of proportionality as a linear relationship in stress–strain curves. Even though significant differences were not observed between Young’s modulus values measured for the samples, a slight tendency to increase this property after the addition of chitosan was observed (Figure 3C). This indicates more rigidity of the samples, probably related to the rising of crystallinity [29] as calculated by DSC measurements. The extract showed no effect in the linear portion of the stress–strain curve.

### 3.5. Color Measurement

**Figure 4:**
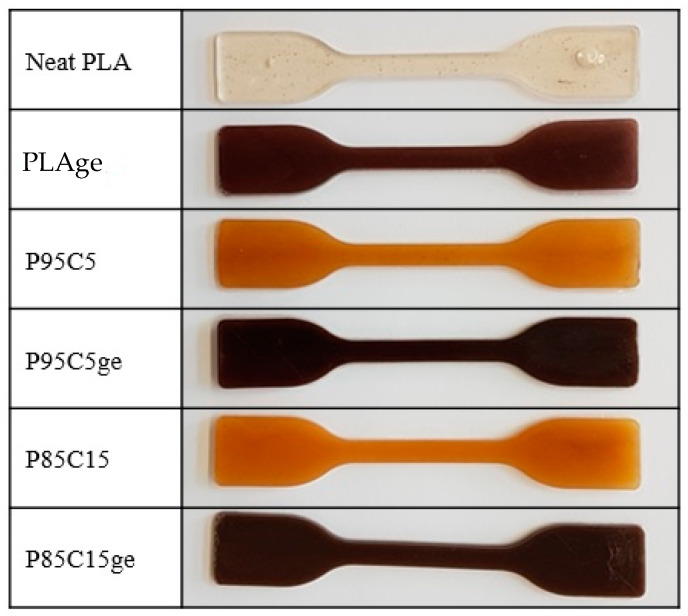
Photograph of the injection-molded test specimens prepared in this study.

Before carrying out the color spectroscopy, the injection-molded test specimens were first photographed (Figure 4). Even under nonstandardized light conditions, it can be clearly seen that adding chitosan at both 5% and 15% leads to a distinct reddish-yellowish tone. After adding the grape seed extract, the test specimens darkened to reddish brown.

In order to evaluate changes in coloration of the samples under standardized conditions, measurements were carried out by using the CIE coordinates values. To assess the effect of chitosan and grape seed extract incorporation on the color properties of the specimen, the parameters L (lightness), a (redness), b (yellowness), and total color difference (ΔE) were measured. As shown in Figure 4, the PLA sample was visually the lightness one, which is consistent with the data obtained by color measurements (Table 4). The PLA control sample showed a higher L* value associated with the brightness characteristic (the white standard used as a background presents the L value of 100).

The incorporation of chitosan decreased the L* parameter and is concentration-dependent, as—for P85C15—less brightness was observed. On the other hand, a considerable change in the b* value was noticed, as adding the polysaccharide shifts it to high values. In fact, the yellowish color is an intrinsic property of chitosan, related to its storage [30] and thermocompression molding [31].

All three samples containing the grape seed extract (GE) were in a similar color range, which is no longer assigned to yellow but to red due to the natural color of the extract. Additionally, the L* parameter decreased in PLAGE, P95C5GE, and P85C15GE samples. In practice, it means that the grape seed extract greatly impacts the color property of the blends, even if it was added in a low ratio.

The color difference (ΔE) of the samples was evaluated in comparison to pure PLA (Table 4). The ΔE was in the range of 40 after the addition of chitosan (P95C5 and P85C15), confirming the color changing behavior of the samples. As mentioned earlier, the high ΔE value can be associated with the thermocompression molding process used in sample preparation and was not significantly dependent on the polysaccharide concentration. As expected, the ΔE for the samples after the inclusion of the GE rose due to the intrinsic characteristic of natural extracts being colorful.

### 3.6. Scanning Electron Microscopy (SEM)

**Figure 5:**
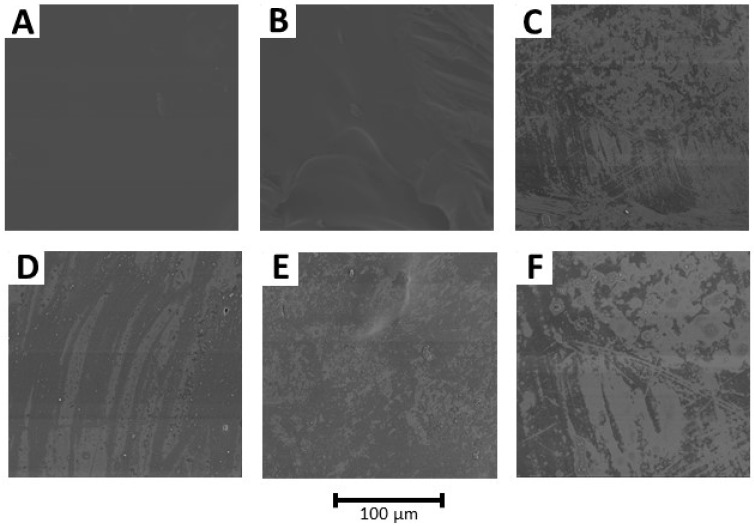
SEM images of the blends. PLA (**A**); P95C5 (**B**); P85C15 (**C**); PLAGE (**D**); P95C5GE (**E**); P85C15GE (**F**). Magnification of 200×.

Characteristic SEM images of pure PLA and blends with and without grape seed extract samples are shown in Figure 5. The surface of the PLA neat sample had a homogeneous structure, and no irregularities were observed (Figure 5A). Oppositely, P95C5 and P85C15 blends showed increased irregularities in their surface appearance (Figure 5B,C), corresponding to the partial miscibility of both polymers.

When GE was incorporated into the blends (Figure 5D–F), a nonhomogeneous surface appearance with the presence of pits was noticed on their surface. Wang et al. [17] described that the addition of grape seed extract resulted in a poorer interfacial adhesion, with weak tensile strength and elongation at break in PCL/chitosan films. In our study, the GE did not affect the mechanical properties, probably due to the presence of chitosan, which acts as a main role in the interfacial adhesion properties, as visualized in elongation at break values, reducing this property to half of the value measured for the PLA neat sample.

## 4. Conclusions

The blends proposed in this study offer an alternative material, not only on the raw source side, but also on the disposal side, via promising end-of-life choices. The extrusion method was chosen to prevent extract aggregation, and homogeneous blends were obtained. Generally, the GE did not affect the mechanical and thermal properties of the blends but added antioxidant properties to the final materials. Chitosan is the main responsible for the reduction in elongation at break and thermal decomposition, mainly due to changes in interfacial adhesion properties. The addition of GSE leads to a nonhomogeneous surface appearance without compromising the mechanical properties of the material. Even though a reduction in elongation at break was observed after the inclusion of chitosan, the production of the proposed blend was achieved with comparable features found the pure PLA.

## Figures and Tables

**Table 1:** Thermal properties of neat PLA and the blends obtained by DSC.

Sample	T_g_ (°C)	T_cc_ (°C)	ΔH_cc_ (J g^−1^)	T_m1_ (°C)	T_m2_ (°C)	ΔH_m_ (J g^−1^)	X_c_ (%)
PLA	58.6	98.2	23.3	147.0	156.4	24.0	25.8
P95C5	56.8	96.8	18.2	146.3	155.8	22.6	25.5
P85C15	56.7/53.0 *	94.2	17.7	144.3	155.0	23.2	29.3
PLAGE	55.9	94.0	16.7	145.3	155.5	16.9	18.2
P95C5GE	53.0	94.5	15.6	144.6	155.2	17.2	19.5
P85C15GE	54.1	93.0	12.9	144.9	155.4	18.9	23.9

* highlighted second glass transition peak for the sample P85C15

**Table 2:** Temperatures at 10%, 25%, 50% and 75% weight loss obtained by TGA.

Sample	T (°C)				Tonset (°C)
	T_10%_	T_25%_	T_50%_	T_75%_	
PLA	287.1	302.0	314.6	329.1	318.7
P95C5	263.2	282.4	298.0	315.1	307.0
P85C15	258.2	277.0	295.5	319.5	292.0
PLAGE	234.7	251.9	265.0	280.4	321.4
P95C5GE	282.5	298.2	311.7	327.4	309.3
P85C15GE	265.4	284.4	302.8	323.5	293.9

**Table 3:** Results of the tensile tests of neat PLA and the blends.

Sample	Tensile Strength (MPa)	Maximum Elongation at Break (%)	Young’s Modulus (MPa)
PLA	55.4 ± 1.8 ^a^	4.1 ± 0.7 ^a^	3306 ± 217 ^a^
P95C5	50.4 ± 4.4 ^a,b^	2.1 ± 0.5 ^b^	3415 ± 258 ^a^
P85C15	49.4 ± 4.1 ^a,b^	2.2 ± 0.8 ^b^	3488 ± 431 ^a^
PLAGE	55.0 ± 0.4 ^a^	3.6 ± 0.5 ^a^	3319 ± 214 ^a^
P95C5GE	47.0 ± 6.0 ^b^	1.5 ± 0.2 ^b^	3582 ± 182 ^a^
P85C15GE	50.5 ± 1.2 ^a,b^	2.0 ± 0.3 ^b^	3808 ± 148 ^a^

The same letter in the column means no significative difference.

**Table 4:** Color measurements values of neat PLA and the blends.

Sample	Color Parameters			
	a*	b*	L*	ΔE
PLA	−0.40	9.43	78.09	0
P95C5	5.33	26.49	46.55	36.32
P85C15	8.38	20.59	40.44	40.24
PLAGE	5.40	3.34	26.53	52.25
P95C5GE	6.69	5.97	28.22	50.49
P85C15GE	5.51	5.05	27.42	51.20

## Data Availability

The datasets generated during the current study are available from the corresponding author upon reasonable request.
